# Associations of Heart Rate Measures during Physical Education with Academic Performance and Executive Function in Children: A Cross-Sectional Study

**DOI:** 10.3390/ijerph17124307

**Published:** 2020-06-16

**Authors:** Adrià Muntaner-Mas, Josep Vidal-Conti, Jo Salmon, Pere Palou-Sampol

**Affiliations:** 1 GICAFE “Physical Activity and Exercise Sciences Research Group”, University of Balearic Islands, 07122 Balearic Islands, Spain; josep.vidal@uib.es (J.V.-C.); pere.palou@uib.es (P.P.-S.); 2 PROFITH “PROmoting FITness and Health through Physical Activity” Research Group, Department of Physical and Sports Education, Sports and Health University Research Institute (iMUDS), Faculty of Sport Sciences, University of Granada, 18071 Granada, Spain; 3 Institute for Physical Activity and Nutrition, School of Exercise and Nutrition Sciences, Deakin University, Burwood, VIC 3125, Australia; jo.salmon@deakin.edu.au

**Keywords:** physical activity, school-aged, cognitive flexibility, inhibition, academic achievement, brain function

## Abstract

The current evidence for a relation between children’s heart rate measures and their academic performance and executive functioning is infancy. Despite several studies observing dose-response effects of physical activity on academic performance and executive function in children, further research using objective measures of the relative intensity of physical activity (e.g., heart rate) is warranted. The present study aimed to inspect associations between heart rate response and various academic performance indicators and executive function domains. A total of 130 schoolchildren between the ages of 9 and 13 years (M = 10.69, SD 0.96 years old; 56.9% boys) participated in a cross-sectional study. Children’s heart rate data were collected through participation in physical education classes using the polar Team^TM^ hardware and software. One week before heart rate measures, academic performance was obtained from the school records in maths, Spanish language, Catalan language, physical education, and Grade point average. Executive function was measured by two domains, cognitive flexibility with the Trail Making Test and inhibition with the Stroop test. Associations between children’s heart rate data and academic performance and executive function were analyzed using regression models. Academic performance was found to be positively related to four heart rate measures (β range, 0.191 to 0.275; all *p* < 0.040). Additionally, the hard heart rate intensity level was positively related to two academic indicators (β range, 0.183 to 0.192; all *p* < 0.044). Three heart rate measures were associated with two cognitive flexibility subdomains (β range, −0.248 to 0.195; all *p* < 0.043), and three heart rate measures were related to one inhibition subdomain (β range, 0.198 to 0.278; all *p* < 0.028). The results showed slight associations of heart rate responses during physical education lessons with academic performance but did not clearly indicate associations with executive function. Future experimental studies testing associations between different bouts of intensity levels are needed to disentangle the relationship with brain function during childhood.

## 1. Introduction

The benefits of physical activity during adolescence are beyond physical health, with evidence from systematic reviews suggesting that participation in physical activity may also enhance brain health in youth [1,2,3]. Studies in neuroscience have found that both acute and chronic physical activity is related to improved brain structure and function [4,5]. Specifically, brain functions, such as cognitive flexibility or inhibition, are higher orders of the executive function and may be enhanced as a result of increased physical activity [6,7,8]. The executive function is closely related to academic performance and considered to be an important prerequisite for successful learning [9]. Arguably, physical activity may benefit executive function which in turn may result in improved academic achievement [2,5,7,10,11,12,13].

Several underlying mechanisms might explain the effects of physical activity on academic performance and executive function. Among them, the cardiorespiratory fitness hypothesis is well documented [2,12]. This idea is supported by the argument that sustained moderate-to-vigorous physical activity enhances cardiorespiratory fitness and this subsequently has positive implications on the brain. Specifically, some research has shown that higher levels of cardiorespiratory fitness are related to increases in angiogenesis and neurogenesis which in turn may lead to improved academic performance and executive function [14,15]. An alternative hypothesis suggests that not all forms of physical activity benefit brain function to the same degree [6,13]. It is hypothesized that cognitively engaging physical activity offers optimal conditions for cognitive enhancement since they jointly involve complex coordinative and cognitive task requirements [16]. This notion is based on the argument that cognitively demanding exercises activate similar brain regions used to control higher-order cognitive processes [17].

In this context, the school environment provides an excellent setting for accumulating moderate-to-vigorous physical activity (which improves cardiorespiratory fitness), particularly, via physical education classes [18,19]. Physical education classes can involve cognitively engaging physical activities, such as team games or motor skill activities, which have been also linked to improvements in executive function [20,21,22]. Thus, physical education provides a unique opportunity to study the relationship between cognitively engaging aerobic activities and academic performance and executive function [23,24].

Furthermore, evidence of a dose–response relationship of physical activity with academic performance and executive function suggests that the intensity of engagement should be accounted for [25,26,27]. A longitudinal study with a large sample size (*n* = 4755) found evidence for a dose–response effect with those children and adolescents doing the most moderate-to-vigorous physical activity having a higher predicted academic performance at adolescence than those doing the least [28]. Castelli et al. [26] concluded that participation in higher physical activity intensities (e.g., vigorous) may have specific benefits on cognitive performance over lower intensities (e.g., moderate) among children. This finding has recently been confirmed by Pindus et al. [25] who found that brief bouts of vigorous physical activity intensity were related to better inhibitory control in children, but not moderate intensities.

The intensity of physical activity in children can be quantified on either absolute (e.g., energy expended) or relative terms (relative to cardiorespiratory fitness). Using relative intensity when tracking physical activity intensity in children is more appropriate than absolute given that the former takes into account cardiorespiratory fitness which is closely related to physical activity [29]. To note, the relative intensity of cardiorespiratory fitness can be expressed in terms of children’s maximal heart rate. In this sense, heart rate (HR) has been historically considered as an objective measure of physical activity intensity in children [30,31,32]. Specifically, HR as an important physiological marker of VO_2_max provides data at the individual level and is suited to categorize children’s’ physical activity intensity [33]. Further, new technological advances in sensors allow HR monitors to measure intensity and time in the HR zones in real-time during defined periods of the day (e.g., during physical education classes). To the best of our knowledge, no previous research has considered relative intensity scales (e.g., heart rate) to study the academic performance and executive function in children.

Therefore, the present study aimed to examine associations between HR measures during physical education classes and academic performance and executive function subdomains in school-aged children from 9 to 13 years old. Given previous research on the association of physical activity with academic performance and executive function, we hypothesized that HR measures would be related to academic performance and executive function.

## 2. Materials and Methods

### 2.1. Study Design and Sample

This cross-sectional design uses baseline data from a convenient sample study based on Spanish (Balearic Islands) schoolchildren between 9 and 13 years of age. All fourth (three class groups), fifth (three class groups) and sixth grade (two-class groups) classes from one public school were invited to participate. A sample of 137 schoolchildren consented to participate in the study and provided data on HR, academic performance, and executive function. Seven participants were excluded because they did not have HR data for at least three physical education classes. In total, a sample of 130 schoolchildren (M = 10.69, SD 0.96 years old; 56.9% boys) was used for this investigation. Data collection was undertaken during the second term of 2019 at school facilities under the supervision of one member of our research group in the presence of physical education teachers. All academic performance and executive function indicators were collected within the previous week before starting the first HR measurement. All participants who provided written informed consent were included in the study. The study was approved by the Human Research Ethics Commission of the University of Balearic Islands and by the school principal (reference number: 108CER19) and abides by the principles set out by the Declaration of Helsinki.

### 2.2. Materials and Measures

#### 2.2.1. Heart Rate Data

The polar Team^TM^ hardware and software (polar Electro, Corp., Finland) were used to objectively collect HR data. This technology enables tracking of HR (in 5-s intervals) of each student in real-time [34]. Specifically, for this investigation, the polar Team App^TM^ (compatible with ipad^TM^) and the polar H10 Heart Sensor^TM^ were used. The polar Team^TM^ allowed the extraction of the following variables during each physical education lesson: minimum HR, average HR, maximum HR, calories burned, total sensor wear time, and time at each HR zone (i.e., very light: 50–60%, light: 60–70%, moderate: 70–80%, hard: 80–90%, very hard: 90–100%). The maximum HR was calculated using the Tanaka equation [35]. The HR range was calculated as the maximum HR minus the minimum HR. Additionally, the Edwards’ TRIMP formulas were used to obtain an internal training load indicator of the HR as (minutes in very hard HR zone × 5) + (minutes in hard HR zone × 4) + (minutes in moderate HR zone × 3) + (minutes in light HR zone × 2) + (minutes in very light HR zone × 1). Generally, unfit children require higher levels of effort than fit ones to do the same activity. To note, all the HR measures indicate the relative intensity of effort performed during physical education lessons.

Before starting the research, a profile of each student was created with the polar App^TM^, including information about age, gender, height, weight, and maximum HR. The HR measures of four complete physical education lessons per each group class were recorded (in total, 32 physical education lessons across all group classes). To note, the 32 physical education lessons were taught for three physical education teachers, one per grade level (i.e., one for fourth, one for fifth and one for the sixth grade) with the same unit content. The Spanish official curricular length of physical education lessons was 60 min, two physical education lessons per week. For analysis purposes, the average HR measures from the valid lessons (at least three) were used.

#### 2.2.2. Academic Performance

Participants’ academic performance was obtained from the school records of the Balearic Island School Register during the same academic year and term when the HR measurements were collected. Specifically, students’ grades (on a scale from 0 to 10) in maths, Spanish language, Catalan language, and physical education were collected. A grade point average (GPA) was also calculated as a single average of the aforementioned subjects.

#### 2.2.3. Executive Function

The executive function was assessed for the domains of cognitive flexibility and inhibition. The Trail Making Test was used as a cognitive flexibility subdomain [36,37]. This test comprises five different conditions (i.e., visual scanning, number sequencing, letter sequencing, number–letter switching, and motor speed). The visual scanning condition assesses the ability to rapidly locate objects. In this condition, the examinee has to scan letters and numbers and mark only the numbers. The number sequencing condition assesses visual-perceptual abilities, and the participants have to draw lines to connect numbers 1–25 in ascending order as fast as possible. The letter sequencing condition assesses basic mental sequencing and letter processing, and the participants have to connect letters in alphabetical order as far as possible. Number–letter switching conditions assess cognitive flexibility and consist of drawing a line to connect the numbers numerically and the letters alphabetically as fast as possible, switching each time from a number to a letter (e.g., 1–A–2–B). Motor speed condition assesses visuomotor speed and consists of drawing a line over the dotted line as quickly as possible. For analysis purposes, a new variable was computed as the time difference between the number sequencing and number–letter switching conditions. Specifically, the total completion time of the number sequencing condition was subtracted from the total completion time of number–letter switching. The difference between number–letter switching and number sequencing was inverted (by multiplying this score by −1) so that a higher score indicates better cognitive flexibility.

To assess inhibition, we used the Stroop test [38]. This test included three conditions and provided a measurement of inhibition (i.e., word reading, color naming, named color-word). The time needed was recorded for all conditions. The word reading condition measures fundamental linguistic skills (i.e., namely speed of naming), and consists in naming the color of filled rectangles. The color naming condition requires participants to name the colors of colored rectangles. The named color-word condition is an indicator of inhibition where color-words are printed in a color that differs from their meaning (e.g., the word “red” printed in green) and the task consists of naming the color of the word (i.e., green in the example) and avoid reading the word. An interference score was obtained by subtracting condition 1 completion time from condition 3 completion time. A smaller Stroop interference score indicates better inhibition, and since we had calculated an inversed Stroop interference score variable by multiplying this score by −1 for analytic purposes, higher scores indicated better inhibition.

#### 2.2.4. Potential Confounders

Age (in years), sex, height, weight, body mass index (BMI), self-reported physical activity levels, physical education teachers, and parental education level were considered as potential confounders. Children in light clothing were weighed twice to the nearest 0.1 kg using a portable electronic scale (TANITA BC 601 Ltd., Paris, France). Height was measured twice to the nearest 0.1 cm without shoes using a portable stadiometer (SECA 213 Ltd., Hamburg, Germany). BMI was calculated by dividing weight (in kilograms) by height squared (in meters). Physical activity levels were self-reported with the Physical Activity Questionnaire for Children (PAQ-C) validated for the Spanish population [39]. The physical education teachers involved in the study were also considered as a potential confounder, in total three teachers participated in this investigation. Children’s parental educational levels were assessed by a self-report questionnaire completed by the mother and father of participants (i.e., “no elementary school”, “elementary school”, “middle school”, “high school”, and “university completed). For analyses, responses were combined as neither parent had a university degree (coded as 1), one parent had a university degree (coded as 2), or both parents had a university degree (coded as 3).

## 3. Statistical Analysis

All outcome variables were checked for normal distribution. Sensitivity analyses showed few significant interactions between sex, and BMI with academic performance indicators, cognitive flexibility, and inhibition; thus, overall, the analyses were performed with the whole sample.

Linear regression models examined associations between HR measures and academic performance, cognitive flexibility, and inhibition. The academic performance indicators, cognitive flexibility, and inhibition domains were included as dependent variables and HR measures as independent variables. Each independent variable (i.e., HR measures) was analyzed in a separate regression model for each dependent variable adjusting for age, sex, physical education teachers, and parental education level. Analyses were additionally adjusted for BMI and physical activity levels with results being minimally changed (data not shown). In sensitive analyses, linear model analyses were conducted with the Restricted Maximum Likelihood Estimation Method. Specifically, the Logarithm of Likelihood −2 (−2LL) was used to ascertain the effects of the grade on dependent variables. The “grade” variable did not prove statistically significant (*p* > 0.05) for any dependent variable, so it was considered that the grade did not affect regression models. Also, the intraclass correlation coefficient (ICC) was estimated for each dependent variable and all the results were virtually zero. We corrected for assessing multiple regressions by defining statistical significance as a Benjamini–Hochberg False Discovery Rate q less than 0.10 [40]. Analyses were performed using the Statistical package for Social Sciences (IBM SpSS Statistics for Windows, version 23.0; IBM Corp, Armonk, NY, USA) and the level of significance was set at *p* < 0.05.

## 4. Results

Descriptive characteristics of the study sample and by sex are presented in Table 1 as means and standard deviations unless otherwise indicated. On average, the distribution of time spent in each intensity was 23.68% in a very light, 31.51% in light, 25.71% in moderate, 15.50% in hard, and 3.60% in very hard HR zones. Table 2 shows the characteristics of heart rate measures and zones for each physical education lesson.

Table 3 shows the associations of each HR measure with academic performance indicators, adjusted for potential confounders. The average HR measure showed a positive and significant association with the Catalan language. The maximum HR was positively related to maths, Catalan language, physical education, and GPA. HR range and Edwards’ TRIMP were positively related to physical Education. The hard HR zone was positively related to physical Education and GPA performance. No significant associations were found for the rest of the variables examined.

Table 4 shows the associations between each HR measure and cognitive flexibility, adjusted for potential confounders. The minimum HR was inversely associated with visual scanning and number sequencing subdomains. The average HR was negatively associated with visual scanning. HR range was positively associated with visual scanning. No significant associations were found for the rest of the variables examined. Table 5 shows the associations between each HR measure with inhibition, adjusted for potential confounders. The minimum, average, and maximum HRs were positively related to color naming. No significant associations were found for the rest of the variables examined.

## 5. Discussion

The overarching objective of this study was to examine associations between different HR measures during physical education lessons with academic performance and executive function in children. Positive relationships were found between four HR measures and four academic performance indicators (except the Spanish Language). Additionally, the hard HR zone was positively related to two academic indicators (Physical Education and GPA). Concerning cognitive flexibility, three HR measures were negatively associated with two cognition subdomains (visual scanning and number sequencing). In terms of inhibition, three HR measures were positively related to one inhibition subdomain (color naming). To our knowledge, our study was unique in that we examined the association between HR during physical education classes (as an objective indicator of physical activity intensity and fitness) with academic performance and executive function.

Our finding that children who worked harder during physical education lessons have better grades in some academic performance indicators suggest intensity could be playing a key role. Previous studies have reported either positive, negative, null, or mediated associations across physical activity intensities with the academic performance [28,41,42,43,44,45,46,47]. However, it is hypothesized that a threshold of physical activity intensity may be needed to benefit brain health [48]. Recent investigations support the notion that a higher exercise intensity induces changes at a molecular level which might result in better academic performance [49]. Furthermore, studies have found moderate-to-vigorous intensity is more strongly related to academic performance than light intensity activity [2,5,7,10,11,12,13]. In our investigation, this finding was not consistent across all HR measures and academic performance indicators. A possible explanation for this discrepancy is that children’s low levels of higher intensity HR might be not sufficient stimulation for academic performance benefits to emerge. It is important to note that children spent an average of 19.4 min (44.81%) of moderate-to-vigorous intensity HR during the physical education lessons. This is consistent with findings by Coe et al. [27], however, in our study only an average of 6.71 min was hard and 1.56 min was categorized as very hard intensity.

Concerning executive function, no clear associations between HR measures were identified with either cognitive flexibility or inhibition. Specifically, inconsistent associations between some HR measures and specific subdomains were identified. Nevertheless, our findings are in agreement with some previous research. As an example, van der Niet et al. [49] reported no clear associations between moderate-to-vigorous physical activity and cognitive flexibility nor inhibition in 80 primary school children aged 8–12 years. Additionally, Mora-Gonzalez et al. [50] showed no relation between objective physical activity with cognitive flexibility nor inhibition in a cross-sectional study involving 100 children with overweight and obesity (10.1 ± 1.1 years old; 58.0% boys).

The current findings are not aligned with intervention studies that have demonstrated executive function improvements from physical activity programs [7,8]. As noted before, the inconsistency between observational and intervention studies should be noted. For instance, Costigan et al. [51] detected small improvements in executive function after 8 weeks of a high-intensity interval training intervention in 65 adolescents. A recent observational study found that a minimum physical activity intensity (e.g., moderate-to-vigorous physical activity) and a specific accumulation pattern are required to provide greater benefits for brain function in children [25]. They found that the number of associations increased with physical activity intensity and with bouts lasting ≥ 30 s. The present study did not examine how the physical intensity was accumulated (i.e., 30 s vs. 60 s bouts) and therefore might not be adequately sensitive to detect differences between physical activity intensities and the executive function domains.

This study is not without limitations. The cross-sectional study design precludes causal inferences. Although potential confounders have been considered in this investigation, physical fitness may mediate the association of physical activity with academic performance and executive function [44]. Nevertheless, we adjusted analyses taking into account self-reported physical activity levels that are directly related to physical fitness components. Further, we did not take into account the time of cognitive exertion during physical activities. Cognitively demanding physical activities seem to have a different effect on children’s brains in comparison with simple aerobic exercises, however, the evidence is still controversial [52,53]. Future research should ascertain differences between simple aerobic exercise vs. cognitively demanding activities for benefits in academic performance and executive function. Additionally, the timing between academic performance and executive function measurements with heart rate data collection should be accounted for as a limitation. In our study, we collected the brain functioning one week before initiating HR measurements; this fact could be different if measurements had been taken immediately after. Likewise, we calculated average data from four physical education lessons, and so the results could be different if more lessons were taken into account. Finally, our measures were unable to differentiate between qualitative aspects of physical activity. For instance, balance or coordinative tasks do not increase HR but might contribute also to improvements in children’s brain health [21,54]. Future research should investigate how different types of exercises affect academic performance and executive function. Lastly, the small sample size should be considered as another limitation.

To the best of our knowledge, this is the first study to assess the cross-sectional relationships between measures of HR during physical education classes and academic performance and executive function. Potential confounders were considered for statistical analyses, as well as adjustments for multiple comparisons.

## 6. Conclusions

This study explored the relationship between children’s HR measures during physical education classes and academic performance and executive function. Firstly, our analyses revealed moderate associations between some HR measures and academic performance indicators. Secondly, we encountered minor associations of HR measures with cognitive flexibility and inhibition. Taken together, our findings suggest that HR measures may be associated with academic performance. In addition, our results were not consistently related to executive function. Our findings call for more investigations on how physical activity is accumulated and the role of specific physical activity intensities in academic performance, and executive function during childhood.

## Figures and Tables

**Table 1:** Descriptive characteristics of the study sample (*n* = 130).

Outcomes	All (*n* = 130)		Fourth Grade (*n* = 51)		Fifth Grade (*n* = 40)		Sixth Grade (*n* = 39)	
	Mean or *n*	SD or %	Mean or *n*	SD or %	Mean or *n*	SD or %	Mean or *n*	SD or %
Age (years)	10.69	0.96	9.78	0.41	10.83	0.59	11.74	0.59
Weight (kg)	39.32	8.94	35.19	7.66	41.18	9.06	42.83	8.40
Height (m)	1.48	0.09	1.41	0.07	1.50	0.08	1.54	0.06
Body mass index (kg/m²)	17.91	2.84	17.70	3.08	18.10	2.65	17.98	2.77
Overweight and obesity (*n*, %)	26	20.00	12	23.50	9	22.50	5	12.80
Parental education university level (*n*, %)	29	22.30	9	17.60	10	25.00	10	25.60
Physical activity levels (PAQ-C)	2.86	0.53	2.72	0.59	2.95	0.50	2.97	0.42
Heart rate measures								
Minimum HR (bpm)	90.06	11.07	92.51	11.28	90.37	9.60	86.53	11.52
Average HR (bpm)	140.44	8.47	142.64	7.77	140.54	7.78	137.46	9.30
Maximum HR (bpm)	190.82	9.43	192.77	7.87	190.71	9.29	188.38	11.00
RangeHR (bpm)	100.77	11.66	100.27	11.70	100.35	10.72	101.85	12.71
Edwards’ TRIMP (min)	105.55	26.52	112.71	25.21	104.14	22.96	97.64	29.57
Calories burned (kcal)	390.50	68.35	414.97	61.18	382.83	57.08	366.38	78.26
Heart rate zones (min × average/lesson)								
Very light (50–60% of HRmax)	10.25	4.17	10.28	4.06	10.21	4.37	10.24	4.22
Light (60–70% of HRmax)	13.64	3.51	13.30	2.65	14.32	4.36	13.38	3.51
Moderate (70–80% of HRmax)	11.13	3.51	12.15	3.21	10.96	3.51	9.98	3.59
Hard (80–90% of HRmax)	6.71	3.73	7.70	3.75	6.41	3.86	5.71	3.33
Very hard (90–100% of HRmax)	1.56	2.19	1.71	2.40	1.35	1.98	1.57	2.15
Moderate + Hard + Very hard	19.40	7.73	21.56	7.29	18.72	7.50	17.26	7.99
Total sensor wear time (min)	43.29	5.83	45.15	5.14	43.26	4.46	40.88	7.05
Academic performance (0–10)								
Maths	6.54	1.41	6.31	1.32	6.90	1.53	6.46	1.35
Spanish language	7.04	1.16	7.02	1.05	7.27	1.18	6.82	1.28
Catalan language	7.17	1.24	7.12	0.97	7.40	1.17	7.00	1.57
Physical education	8.12	1.03	8.20	0.75	8.05	1.01	8.10	1.33
GPA	7.22	0.92	7.16	0.70	7.41	0.90	7.10	1.16
Executive function								
Cognitive flexibility (s)								
Trail Making Test								
Condition 1: Visual Scanning	69.08	7.69	69.43	6.47	71.25	6.43	66.41	9.53
Condition 2: Number Sequencing	60.11	8.63	59.90	8.28	60.08	9.34	60.41	8.53
Condition 3: Letter Sequencing	68.90	6.42	69.02	6.20	70.58	5.57	67.03	7.13
Condition 4: Number–Letter Switching	140.58	39.91	134.02	38.53	154.85	33.24	134.51	44.83
Condition 5: Motor Speed	63.02	9.14	64.73	7.76	63.95	8.20	59.82	10.96
4-2 (condition time difference) ^‡^	80.47	36.52	74.12	36.14	94.78	29.22	74.10	40.25
Inhibition (s)								
Stroop Test								
Condition 1: Word reading	69.28	9.11	70.43	8.88	66.60	7.54	70.54	10.43
Condition 2: Color naming	53.15	11.26	51.75	9.74	53.10	12.68	55.03	11.58
Condition 3: Named color-word	28.02	7.72	28.51	6.93	28.20	9.39	27.21	6.90
Interference score (3-1 conditions) ^‡^	41.26	10.41	41.92	9.72	38.40	12.43	43.33	8.45

Values are mean (± SD) or percentages, unless otherwise indicated. Bpm: beats per min; HR: heart rate; HRmax: maximum heart rate; GPA: grade point average; W: word; C: color; CW: color-word. **^‡^** Lower values indicate better results.

**Table 2:** Descriptive characteristics of heart rate measures for each physical education lesson (*n* = 130).

Physical Education Lessons	1		2		3		4	
Heart rate measures								
Minimum HR (bpm)	86.97	21.30	90.35	16.00	86.78	15.85	79.65	34.23
Average HR (bpm)	134.75	20.87	140.61	13.73	139.30	10.85	122.19	49.56
Maximum HR (bpm)	182.52	25.52	190.86	16.90	191.82	10.86	164.73	66.54
Range HR (bpm)	95.55	21.62	100.51	18.13	105.05	16.33	85.08	37.07
Edwards’ TRIMP (min)	82.35	42.28	106.66	38.78	107.28	31.77	98.15	53.57
Calories burned (kcal)	322.81	115.01	396.82	100.28	399.94	83.43	351.06	170.28
Heart rate zones (min × average/lesson)								
Very light (50–60% of HRmax)	11.21	6.59	9.02	6.13	10.90	5.80	7.43	6.40
Light (60–70% of HRmax)	12.03	5.52	13.24	5.43	14.22	5.06	11.49	7.46
Moderate (70–80% of HRmax)	8.58	5.83	10.93	5.08	11.45	4.59	10.50	6.51
Hard (80–90% of HRmax)	4.41	4.91	7.24	4.83	6.58	4.58	6.72	6.03
Very hard (90–100% of HRmax)	0.74	1.86	1.88	3.01	1.45	3.06	1.87	3.90
Moderate + Hard + Very hard	13.73	3.93	20.05	4.55	19.48	5.00	19.09	4.33
Total sensor wear time (min)	36.98	13.07	42.13	11.61	44.60	7.22	38.01	17.49

Bpm: beats per min; HR: heart rate; HRmax: maximum heart rate.

**Table 3:** Associations of HR measures with academic performance indicators (*n* = 130).

Academic Performance	Maths		Spanish Language		Catalan Language		Physical Education		GPA	
	*β*	*p*	*β*	*p*	*β*	*p*	*β*	*p*	*β*	*p*
HR measures										
Minimum HR (bpm)	0.001	0.823	0.078	0.392	0.096	0.299	−0.054	0.559	0.050	0.589
Average HR (bpm)	0.145	0.120	0.151	0.105	0.197	**0.034**	0.079	0.404	0.191	**0.040**
Maximum HR (bpm)	0.229	**0.012 ^‡^**	0.171	0.063	0.232	**0.012 ^‡^**	0.201	**0.029**	0.275	**0.002 ^‡^**
Range HR (bpm)	0.153	0.082	0.058	0.512	0.089	0.321	0.199	**0.025**	0.162	0.067
Edwards’ TRIMP (min)	0.080	0.386	0.124	0.180	0.107	0.247	0.195	**0.035**	0.160	0.083
Calories burned (kcal)	0.071	0.450	0.110	0.244	0.108	0.255	0.174	0.066	0.147	0.121
HR zones (min)										
Very light (50–60% of HRmax)	−0.162	0.067	−0.146	0.102	−0.115	0.200	−0.035	0.696	−0.156	0.079
Light (60–70% of HRmax)	0.007	0.933	0.083	0.348	0.078	0.384	0.030	0.741	0.063	0.476
Moderate (70–80% of HRmax)	0.041	0.660	0.081	0.383	0.094	0.309	0.082	0.374	0.095	0.301
Hard (80–90% of HRmax)	0.099	0.277	0.169	0.064	0.115	0.212	0.192	**0.035**	0.183	**0.044**
Very hard (90–100% of HRmax)	0.075	0.410	−0.012	0.896	0.002	0.986	0.114	0.213	0.057	0.532
Moderate + Hard + Very hard	0.089	0.335	0.116	0.208	0.099	0.283	0.164	0.074	0.149	0.104

The β values are standardized. These analyses were adjusted for the following covariates: sex, age, parental educational level, and physical education teachers. The bold font is used to highlight the significance level at *p* < 0.05. Bpm: beats per min; HR: heart rate; HRmax: maximum heart rate; GPA: grade point average. ^‡^ These associations remained significant after adjustment for multiple comparisons using the Benjamini and Hochberg method. The academic performance indicators were taken one week before heart rate measures.

**Table 4:** Associations of HR measures with cognitive flexibility subdomains (*n* = 130).

Cognitive Flexibility	Visual Scanning		Number Sequencing		Letter Sequencing		Number–Letter Switching		Motor Speed		4-2 (Condition Time Difference) *	
	*β*	*p*	*β*	*p*	*β*	*p*	*β*	*p*	*β*	*p*	*β*	*p*
HR measures												
Minimum HR (bpm)	−0.248	**0.006 ^‡^**	−0.200	**0.027**	−0.089	0.329	−0.150	0.100	−0.053	0.551	−0.117	0.202
Average HR (bpm)	−0.188	**0.043**	−0.149	0.109	−0.039	0.671	−0.161	0.085	−0.041	0.653	−0.140	0.134
Maximum HR (bpm)	−0.035	0.706	−0.023	0.801	0.036	0.692	−0.102	0.268	−0.009	0.925	−0.106	0.251
Range HR (bpm)	0.195	**0.027**	0.161	0.068	0.106	0.227	0.057	0.520	0.041	0.635	0.025	0.783
Edwards’ TRIMP (min)	−0.009	0.924	−0.101	0.272	0.028	0.758	−0.033	0.726	0.113	0.212	−0.012	0.900
Calories burned (kcal)	−0.035	0.710	−0.101	0.282	0.017	0.855	−0.066	0.490	0.094	0.310	−0.048	0.617
HR zones (min)												
Very light (50–60% of HRmax)	−0.019	0.836	0.094	0.293	−0.026	0.766	0.024	0.790	−0.052	0.548	0.044	0.965
Light (60–70% of HRmax)	−0.098	0.268	−0.079	0.373	−0.122	0.165	−0.019	0.831	−0.017	0.844	−0.002	0.981
Moderate (70–80% of HRmax)	0.075	0.419	−0.095	0.303	0.054	0.553	0.054	0.556	0.132	0.141	0.082	0.377
Hard (80–90% of HRmax)	0.028	0.757	−0.087	0.338	0.071	0.430	−0.018	0.841	0.090	0.313	0.000	0.996
Very hard (90–100% of HRmax)	−0.056	0.540	−0.015	0.872	0.011	0.900	−0.100	0.275	0.051	0.570	−0.150	0.250
Moderate + Hard + Very hard	0.032	0.732	−0.090	0.328	0.063	0.491	−0.013	0.887	0.119	0.186	0.007	0.941

The β values are standardized. These analyses were adjusted for the following covariates: sex, age, parental educational level, and physical education teachers. The bold font is used to highlight the significance level at *p* < 0.05. Bpm: beats per min; HR: heart rate; HRmax: maximum heart rate. * The values were inverted so that higher values indicate better results. ^‡^ These associations remained significant after adjustment for multiple comparisons using the Benjamini and Hochberg method. The cognitive flexibility subdomains were taken one week before heart rate measures.

**Table 5:** Associations of HR measures with inhibition subdomains (*n* = 130).

Inhibition	Word Reading		Color Naming		Named Color-Word		Interference Score (3-1 Conditions) *	
	*β*	*p*	*β*	*p*	*β*	*p*	*β*	*p*
HR measures								
Minimum HR (bpm)	0.155	0.091	0.198	**0.028**	0.054	0.558	0.096	0.300
Average HR (bpm)	0.100	0.288	0.278	**0.002** **^‡^**	0.040	0.669	0.057	0.543
Maximum HR (bpm)	−0.010	0.915	0.252	**0.005** **^‡^**	0.006	0.946	−0.013	0.887
Range HR (bpm)	−0.146	0.101	0.013	0.886	−0.044	0.626	−0.095	0.287
Edwards’ TRIMP (min)	−0.043	0.643	0.064	0.483	−0.021	0.823	−0.022	0.811
Calories burned (kcal)	−0.005	0.961	0.057	0.547	−0.028	0.770	0.017	0.862
HR zones (min)								
Very light (50–60% of HRmax)	0.025	0.779	−0.105	0.236	−0.026	0.772	0.041	0.646
Light (60–70% of HRmax)	0.085	0.338	0.026	0.764	0.026	0.775	0.056	0.532
Moderate (70–80% of HRmax)	−0.037	0.692	−0.004	0.967	−0.078	0.401	0.026	0.783
Hard (80–90% of HRmax)	−0.084	0.362	0.049	0.591	0.002	0.986	−0.074	0.419
Very hard (90–100% of HRmax)	−0.021	0.820	0.113	0.212	0.015	0.868	−0.030	0.748
Moderate + Hard + Very hard	−0.064	0.491	0.055	0.550	−0.030	0.746	−0.033	0.719

The β values are standardized. These analyses were adjusted for the following covariates: sex, age, parental educational level, and physical education teachers. The bold font is used to highlight the significance level at *p* < 0.05. * The values were inverted so that higher values indicate better results. Bpm: beats per min; HR: heart rate; HRmax: maximum heart rate; ^‡^ These associations remained significant after adjustment for multiple comparisons using the Benjamini and Hochberg method. The inhibition subdomains were taken one week before heart rate measures.

## References

[1] Rodriguez-Ayllon M., Cadenas-Sánchez C., Estévez-López F., Muñoz N.E., Mora-Gonzalez J., Migueles J.H., Molina-García P., Henriksson H., Mena-Molina A., Martínez-Vizcaíno V. (2019). Role of physical Activity and Sedentary Behavior in the Mental Health of preschoolers, Children and Adolescents: A Systematic Review and Meta-Analysis. Sport Med..

[2] Erickson K.I., Hillman C., Stillman C.M., Ballard R.M., Bloodgood B., Conroy D.E., Macko R., Marquez D.X., Petruzzello S.J., Powell K.E. (2019). Physical Activity, Cognition, and Brain Outcomes. Med. Sci. Sport Exerc..

[3] Lubans D., Richards J., Hillman C., Faulkner G., Beauchamp M., Nilsson M., Kelly P., Smith J., Raine L., Biddle S. (2016). Physical Activity for Cognitive and Mental Health in Youth: A Systematic Review of Mechanisms. Pediatrics.

[4] Valkenborghs S.R., Noetel M., Hillman C.H., Nilsson M., Smith J.J., Ortega F.B., Lubans D.R. (2019). The Impact of physical Activity on Brain Structure and Function in Youth: A Systematic Review. Pediatrics.

[5] Hillman C., Logan N., Shigeta T. (2019). A Review of Acute physical Activity Effects on Brain and Cognition in Children. Transl. J. Am. Coll. Sport Med..

[6] Best J.R. (2010). Effects of physical Activity on Children’s Executive Function: Contributions of Experimental Research on Aerobic Exercise. Dev. Rev..

[7] De Greeff J.W., Bosker R.J., Oosterlaan J., Visscher C., Hartman E. (2018). Effects of physical activity on executive functions, attention and academic performance in preadolescent children: A meta-analysis. J. Sci. Med. Sport.

[8] Verburgh L., Königs M., Scherder E.J.A., Oosterlaan J. (2014). Physical exercise and executive functions in preadolescent children, adolescents and young adults: A meta-analysis. Br. J. Sports Med..

[9] Cortés Pascual A., Moyano Muñoz N., Quílez Robres A. (2019). The Relationship Between Executive Functions and Academic performance in primary Education: Review and Meta-Analysis. Front. Psychol..

[10] Biddle S.J.H., Ciaccioni S., Thomas G., Vergeer I. (2019). Physical activity and mental health in children and adolescents: An updated review of reviews and an analysis of causality. Psychol. Sport Exerc..

[11] Singh A.S., Saliasi E., van den Berg V., Uijtdewilligen L., de Groot R.H.M., Jolles J., Andersen L.B., Bailey R., Chang Y.-K., Diamond A. (2019). Effects of physical activity interventions on cognitive and academic performance in children and adolescents: A novel combination of a systematic review and recommendations from an expert panel. Br. J. Sports Med..

[12] Marques A., Santos D.A., Hillman C.H., Sardinha L.B. (2018). How does academic achievement relate to cardiorespiratory fitness, self-reported physical activity and objectively reported physical activity: A systematic review in children and adolescents aged 6–18 years. Br. J. Sports Med..

[13] Vazou S., Pesce C., Lakes K., Smiley-Oyen A. (2019). More than one road leads to Rome: A narrative review and meta-analysis of physical activity intervention effects on cognition in youth. Int. J. Sport Exerc. Psychol..

[14] Hillman C.H., Erickson K.I., Kramer A.F. (2008). Be smart, exercise your heart: Exercise effects on brain and cognition. Nat. Rev. Neurosci..

[15] Van praag H. (2008). Neurogenesis and Exercise: Past and Future Directions. Neuromol. Med..

[16] Pesce C., Croce R., Ben-Soussan T.D., Vazou S., McCullick B., Tomporowski P.D., Horvat M. (2019). Variability of practice as an interface between motor and cognitive development. Int. J. Sport Exerc. Psychol..

[17] Diamond A., Lee K. (2011). Interventions Shown to Aid Executive Function Development in Children 4 to 12 Years Old. Science.

[18] Kerr C., Smith L., Charman S., Harvey S., Savory L., Fairclough S., Govus A. (2018). physical education contributes to total physical activity levels and predominantly in higher intensity physical activity categories. Eur. Phys. Educ. Rev..

[19] Silva D.A.S., Chaput J.-P., Katzmarzyk P.T., Fogelholm M., Hu G., Maher C., Olds T., Onywera V., Sarmiento O.L., Standage M. (2018). Physical Education Classes, physical Activity, and Sedentary Behavior in Children. Med. Sci. Sport Exerc..

[20] Schmidt M., Jäger K., Egger F., Roebers C.M., Conzelmann A. (2015). Cognitively Engaging Chronic physical Activity, But Not Aerobic Exercise, Affects Executive Functions in primary School Children: A Group-Randomized Controlled Trial. J. Sport Exerc. Psychol..

[21] Van der Fels I.M.J., Smith J., de Bruijn A.G.M., Bosker R.J., Königs M., Oosterlaan J., Visscher C., Hartman E. (2019). Relations between gross motor skills and executive functions, controlling for the role of information processing and lapses of attention in 8–10 year old children. PLoS ONE.

[22] Rudd J.R., O’Callaghan L., Williams J. (2019). Physical Education pedagogies Built upon Theories of Movement Learning: How Can Environmental Constraints Be Manipulated to Improve Children’s Executive Function and Self-Regulation Skills?. Int. J. Environ. Res. Public Health.

[23] Carlson S.A., Fulton J.E., Lee S.M., Maynard L.M., Brown D.R., Kohl H.W., Dietz W.H. (2008). Physical Education and Academic Achievement in Elementary School: Data From the Early Childhood Longitudinal Study. Am. J. Public Health.

[24] Rasberry C.N., Lee S.M., Robin L., Laris B.A., Russell L.A., Coyle K.K., Nihiser A.J. (2011). The association between school-based physical activity, including physical education, and academic performance: A systematic review of the literature. Prev. Med. (Balt.).

[25] Pindus D.M., Drollette E.S., Raine L.B., Kao S.-C., Khan N., Westfall D.R., Hamill M., Shorin R., Calobrisi E., John D. (2019). Moving fast, thinking fast: The relations of physical activity levels and bouts to neuroelectric indices of inhibitory control in preadolescents. J. Sport Heal. Sci..

[26] Castelli D.M., Hillman C.H., Hirsch J., Hirsch A., Drollette E. (2011). FIT Kids: Time in target heart zone and cognitive performance. Prev. Med. (Balt.).

[27] Coe D.P., Pivarnik J.M., Womack C.J., Reeves M.J., Malina R.M. (2006). Effect of physical Education and Activity Levels on Academic Achievement in Children. Med. Sci. Sport Exerc..

[28] Booth J.N., Leary S.D., Joinson C., Ness A.R., Tomporowski P.D., Boyle J.M., Reilly J.J. (2014). Associations between objectively measured physical activity and academic attainment in adolescents from a UK cohort. Br. J. Sports Med..

[29] Piercy K.L., Troiano R.P., Ballard R.M., Carlson S.A., Fulton J.E., Galuska D.A., George S.M., Olson R.D. (2018). The Physical Activity Guidelines for Americans. JAMA.

[30] Sirard J.R., Pate R.R. (2001). Physical Activity Assessment in Children and Adolescents. Sport Med..

[31] Freedson P.S., Miller K. (2000). Objective monitoring of physical activity using motion sensors and heart rate. Res. Q. Exerc. Sport.

[32] Bassett D.R. (2000). Validity and reliability issues in objective monitoring of physical activity. Res. Q. Exerc. Sport.

[33] Eckard M.L., Kuwabara H.C., Van Camp C.M. (2019). Using heart rate as a physical activity metric. J. Appl. Behav. Anal..

[34] Gilgen-Ammann R., Schweizer T., Wyss T. (2019). RR interval signal quality of a heart rate monitor and an ECG Holter at rest and during exercise. Eur. J. Appl. Physiol..

[35] Cicone Z.S., Holmes C.J., Fedewa M.V., MacDonald H.V., Esco M.R. (2019). Age-Based prediction of Maximal Heart Rate in Children and Adolescents: A Systematic Review and Meta-Analysis. Res. Q. Exerc. Sport.

[36] Sánchez-Cubillo I., Periáñez J.A., Adrover-Roig D., Rodríguez-Sánchez J.M., Ríos-Lago M., Tirapu J., Barceló F. (2009). Construct validity of the Trail Making Test: Role of task-switching, working memory, inhibition/interference control, and visuomotor abilities. J. Int. Neuropsychol. Soc..

[37] Swanson J. (2005). The Delis-Kaplan Executive Function System: A Review. Can. J. Sch. Psychol..

[38] Scarpina F., Tagini S. (2017). The Stroop Color and Word Test. Front. Psychol..

[39] Benítez-porres J., López-Fernández I., Raya J.F., Álvarez Carnero S., Alvero-Cruz J.R., Álvarez Carnero E. (2016). Reliability and Validity of the PAQ-C Questionnaire to Assess physical Activity in Children. J. Sch. Health.

[40] Benjamini Y., Hochberg Y. (1995). Controlling the False Discovery Rate: A practical and powerful Approach to Multiple Testing. J. R. Stat. Soc. Ser. B.

[41] Lima R.A., Pfeiffer K.A., Møller N.C., Andersen L.B., Bugge A. (2019). Physical Activity and Sedentary Time Are Positively Associated With Academic Performance: A 3-Year Longitudinal Study. J. Phys. Act. Heal..

[42] Esteban-Cornejo I., Tejero-González C.M., Martinez-Gomez D., Cabanas-Sánchez V., Fernández-Santos J.R., Conde-Caveda J., Sallis J.F., Veiga O.L. (2014). Objectively measured physical activity has a negative but weak association with academic performance in children and adolescents. Acta Paediatr..

[43] Kwak L., Kremers S.P.J., Bergman P., Ruiz J.R., Rizzo N.S., Sjöström M. (2009). Associations between physical activity, fitness, and academic achievement. J. Pediatr..

[44] Kyan A., Takakura M., Miyagi M. (2019). Mediating effect of aerobic fitness on the association between physical activity and academic achievement among adolescents: A cross-sectional study in Okinawa, Japan. J. Sports Sci..

[45] LeBlanc M.M., Martin C.K., Han H., Newton R., Sothern M., Webber L.S., Davis A.B., Williamson D.A. (2012). Adiposity and Physical Activity Are Not Related to Academic Achievement in School-Aged Children. J. Dev. Behav. Pediatr..

[46] Maher C., Lewis L., Katzmarzyk P.T., Dumuid D., Cassidy L., Olds T. (2016). The associations between physical activity, sedentary behaviour and academic performance. J. Sci. Med. Sport.

[47] Pindus D.M., Drollette E.S., Scudder M.R., Khan N.A., Raine L.B., Sherar L.B., Esliger D.W., Kramer A.F., Hillman C.H. (2016). Moderate-to-vigorous physical activity, indices of cognitive control, and academic achievement in preadolescents. J. Pediatr..

[48] Herold F., Müller P., Gronwald T., Müller N.G. (2019). Dose–Response Matters! A perspective on the Exercise prescription in Exercise-Cognition Research. Front. Psychol..

[49] Van der Niet A.G., Smith J., Scherder E.J.A., Oosterlaan J., Hartman E., Visscher C. (2015). Associations between daily physical activity and executive functioning in primary school-aged children. J. Sci. Med. Sport.

[50] Mora-Gonzalez J., Esteban-Cornejo I., Cadenas-Sanchez C., Migueles J.H., Molina-Garcia P., Rodriguez-Ayllon M., Henriksson P., Pontifex M.B., Catena A., Ortega F.B. (2019). Physical Fitness, Physical Activity, and the Executive Function in Children with Overweight and Obesity. J. Pediatr..

[51] Costigan S.A., Eather N., Plotnikoff R.C., Hillman C.H., Lubans D.R. (2016). High-Intensity Interval Training for Cognitive and Mental Health in Adolescents. Med. Sci. Sport Exerc..

[52] Hillman C.H., McAuley E., Erickson K.I., Liu-Ambrose T., Kramer A.F. (2019). On mindful and mindless physical activity and executive function: A response to Diamond and Ling (2016). Dev. Cogn. Neurosci..

[53] Diamond A., Ling D.S. (2019). Aerobic-Exercise and resistance-training interventions have been among the least effective ways to improve executive functions of any method tried thus far. Dev. Cogn. Neurosci..

[54] Fernandes V.R., Ribeiro M.L.S., Melo T., de Tarso Maciel-Pinheiro P., Guimarães T.T., Araújo N.B., Ribeiro S., Deslandes A.C. (2016). Motor Coordination Correlates with Academic Achievement and Cognitive Function in Children. Front. Psychol..

